# Inspection Robot Navigation Based on Improved TD3 Algorithm

**DOI:** 10.3390/s24082525

**Published:** 2024-04-15

**Authors:** Bo Huang, Jiacheng Xie, Jiawei Yan

**Affiliations:** School of Mechanical Engineering, Sichuan University of Science and Engineering, Zigong 643099, China

**Keywords:** inspection robot navigation, deep reinforcement learning, long- and short-term memory, curiosity-driven

## Abstract

The swift advancements in robotics have rendered navigation an essential task for mobile robots. While map-based navigation methods depend on global environmental maps for decision-making, their efficacy in unfamiliar or dynamic settings falls short. Current deep reinforcement learning navigation strategies can navigate successfully without pre-existing map data, yet they grapple with issues like inefficient training, slow convergence, and infrequent rewards. To tackle these challenges, this study introduces an improved two-delay depth deterministic policy gradient algorithm (LP-TD3) for local planning navigation. Initially, the integration of the long–short-term memory (LSTM) module with the Prioritized Experience Re-play (PER) mechanism into the existing TD3 framework was performed to optimize training and improve the efficiency of experience data utilization. Furthermore, the incorporation of an Intrinsic Curiosity Module (ICM) merges intrinsic with extrinsic rewards to tackle sparse reward problems and enhance exploratory behavior. Experimental evaluations using ROS and Gazebo simulators demonstrate that the proposed method outperforms the original on various performance metrics.

## 1. Introduction

Continuous breakthroughs in key robotics technologies have made autonomous mobile robots a focal point in intelligent robotics research. Currently, autonomous mobile robots are widely used in transportation, industry, agriculture, the service sector, and aerospace, and are capable of performing tasks such as patrolling, rescue, logistics, transportation, and planetary exploration [1,2]. Thus, a robust autonomous navigation system is crucial for the deployment of intelligent robots, enabling them to avoid collisions with dynamic and static obstacles, safely reach the target point via an optimal or suboptimal route, and complete assigned subsystem tasks.

Navigation, which encompasses both global and local aspects [3], is a critical task in autonomous mobile robot research. Since the 1980s, researchers have extensively explored and studied navigation planning for autonomous mobile robots, yielding numerous mature findings. In 1968, Hart et al. [4] developed the A* algorithm, an enhancement of Dijkstra’s method [5] for finding the shortest paths in graphs or networks. A* distinguishes itself by incorporating a heuristic function, a mechanism that intelligently guides the selection of the next node during the search process, unlike Dijkstra’s algorithm. In 1994, Kavraki et al. [6] introduced the Probabilistic Roadmap Method (PRM), a sampling-based algorithm for robot motion planning aimed at efficiently identifying collision-free paths within complex, high-dimensional environments. Following this, researchers have proposed improvements to original methods, achieving more refined navigation planning techniques. Shentu et al. [7] developed a hybrid navigation control system using a motion controller designed via the backpropagation method and incorporated Kalman filtering to minimize localization errors. The system adapts to various guidance sensors across different distances. Yufeng Li et al. [8] enhanced navigation accuracy and stability by combining an improved A* algorithm with the Dynamic Window Approach (DWA) for global and local path planning, outperforming traditional fusion algorithms. Additionally, Chien-Yen Wang et al. [9] introduced a partition-based map representation to augment the A* path planner (PBPP), effectively narrowing the search space and reducing time consumption, leading to more efficient path planning. The classical approach currently combines various algorithms, like SLAM (Simultaneous Localization and Mapping), to convert perceived environmental information into a global map for path-planning modules. Based on global and local path planning results, the control module dynamically adjusts speed, direction, and other parameters to guide the robot toward the target. In summary, traditional navigation frameworks depend on high-precision global maps, necessitating costly sensors and manual calibration to meet requirements. Additionally, the integrated computation in traditional navigation frameworks leads to cumulative errors, and the high sensitivity of the sensors to noise significantly reduces the effectiveness of these methods in complex, dynamic, or unknown environments [10,11].

The rapid advancement in deep reinforcement learning techniques and hardware has drawn increasing attention from researchers. Among these, learning-based methods have become a research focus, achieving significant success across diverse fields. In 2013, Mnih et al. [12] introduced the first deep learning model capable of learning control strategies directly from high-dimensional perceptual input, achieving success in this endeavor. Applying this model to the Atari 2600 game, they found it outperformed human experts. In 2015, David Silver et al. [13] developed the Double Deep Q-Network (DDQN) to address the issue of overestimation of DQN, with experimental results that indicate improved performance. In 2016, to address the limitations with continuous actions, Timothy P. Lillicrap et al. [14] introduced the Model-free Deterministic Policy Gradient-Based Algorithm (DDPG), which showed promising results in various simulation control scenarios. Subsequent studies introduced algorithms like the Asynchronous Dominant Action Evaluation (A3C) [15], Proximal Policy Optimization (PPO) [16], Two-Delay Deep Deterministic Policy Gradient (TD3) [17], and Deep Spiking Q-Network (DSQN) [18]. These frameworks aim to solve issues such as slow convergence, low model robustness, and weak generalization during training.

Deep reinforcement learning-based schemes, with their powerful characterization capabilities and excellence in handling high-dimensional dynamic scenarios (e.g., LiDAR, images) have been introduced to intelligent robot navigation research. Deep reinforcement learning offers a flexible, adaptive, and efficient way to solve robot navigation problems by learning decision strategies or value functions directly from raw data, without the need for human intervention or feature engineering. The end-to-end model enables autonomous robot–environment interaction for learning navigation strategies without depending on predefined rules or map information. Through the trained model, the robot makes real-time decisions in a mapless environment, enabling it to navigate and perform inspection tasks in unknown environments effectively.

## 2. Related Work

SLAM technology, comprising laser SLAM and visual SLAM, utilizes laser and visual sensors, respectively, to achieve real-time localization and mapping in unknown environments, facilitating autonomous robot navigation. Visual SLAM depends on precise image features but is affected by lighting, sensor parameters, and environmental changes. Laser SLAM, requiring high-precision LiDAR, suffers from low computational efficiency and large cumulative errors in extensive environments, limiting its application. Wang, X et al. [19] proposed a SLAM application system based on the fusion of multi-line LiDAR and visual perception, tailored to sensor characteristics and application scenarios, incorporating a hybrid path-planning algorithm that combines the A* and Time Elastic Band (TEB) algorithms. Experimental results indicate that the designed SLAM and path planning methods exhibit good reliability and stability.

In the realm of learning-based strategies, Piotr Mirowski et al. [20] developed an agent capable of navigating complex 3D mazes by integrating goal-driven reinforcement learning with auxiliary depth prediction and loop-closure classification tasks, achieving human-level performance even with frequent changes in the target location. Lei Tai et al. [21] developed a mapless motion planner using deep reinforcement learning, dubbed ADDPG. They separated the sample collection process into another thread, achieving parallel training through multiple threads. This planner accepts 10-dimensional sparse laser data and the target location in the robot’s relative coordinate system as inputs, requiring only minor adjustments for direct application in unknown real-world environments. Yuke Zhu et al. [22] introduced a deep reinforcement learning framework tailored for goal-driven visual navigation. Uniquely, this framework treats task objectives as model inputs, avoiding the integration of goals directly into the model’s parameters. This methodology effectively tackles the problem of generalizing across varied scenes and objectives. Furthermore, they presented the AI2-THOR framework, which supports economical and efficient data collection. Linhai Xie et al. [23] proposed the Dueling DQN approach (D3QN), inspired by the Fully Convolutional Residual Networks (FCRN) [24] for predicting depth information from RGB images. The network, trained in a simulator using monocular RGB images as an input, successfully transfers the model from virtual to real-world settings, with D3QN outperforming traditional DQN methods.

The primary aim of this research is to equip robots with navigation skills through learning-based strategies, focusing on enhancing the learning efficiency and generalization of DRL models in subsequent studies. Junli Gao et al. [25] merged the PRM algorithm with TD3, employing incremental training to fast-track learning and notably enhance the model’s generalization ability. Lu Chang et al. [26] integrated local path planning with deep reinforcement learning to develop an enhanced DWA [27] based on Q-learning. They refined the original evaluation function to increase the scores of better trajectories in each specific sub-function and introduced two additional sub-functions to accommodate more complex scenarios, thus improving global navigation performance. Hartmut Surmann et al. [28] developed a fast parallel robot simulation environment, addressing the problem of slow neural network learning speeds in existing simulation frameworks. Meiqiang Zhu et al. [29] proposed a universal reward function based on Matching Networks (MN) to address the issue of sparse rewards. This function facilitates reward shaping from similar navigation task trajectories without human supervision, thus accelerating the training speed of DRL for new tasks. Lastly, Reinis Cimurs et al. [30] developed an autonomous navigation system based on the double-delayed deep deterministic policy gradient algorithm, utilizing perceptual data to identify points of interest and select optimal waypoints. This system, trained through deep reinforcement learning, facilitates autonomous navigation from waypoints to global objectives without any prior map information, achieving superior navigation performance in complex static and dynamic environments.

This study aims to improve dual-delay deep deterministic policy gradient algorithms for autonomous robot navigation by better utilizing empirical data, speeding up convergence, and addressing reward sparsity. LiDAR data was chosen for training due to their simplicity, reduced resource demands relative to camera data, and their advantages in real-time performance, stability, and deployment in diverse settings.

The main contributions of the research are summarized as follows:

(1) We propose a training framework that incorporates temporal navigation experience by integrating the long–short-term memory module (LSTM) with Prioritized Experience Replay (PER) into the TD3 network structure, enhancing sample data utilization, and speeding up the learning process.
(2) We introduce an Intrinsic Curiosity Module (ICM) to the dual-delay deep deterministic policy gradient algorithm’s reward system to address reward sparsity and boost exploration capabilities.
(3) We propose a new state representation that incorporates laser sensor data, the robot’s current state, and the distance to the target point as input during model training, thus enabling adaptation to various environments.

The remainder of this paper is organized as follows: Section 3, Part 1 introduces the DDPG algorithm within deep reinforcement learning methods and the background of the TD3 algorithm. Section 3, Part 2 details the mapless robot navigation approach utilizing the Long-Short-Term Memory Module and the Prioritized Experience Playback mechanism (LP-TD3). Section 3, Part 3 explores the application of curiosity-driven exploration strategies in robot navigation. Section 4 will present simulation experiment results and analysis. Finally, Section 5 will conclude and outline future research directions.

## 3. Materials and Methods

### 3.1. Algorithmic Background

Reinforcement learning is an approach where an agent learns to make decisions by maximizing rewards through interaction with its environment, modeled as Markov Decision Processes (MDPs) comprising state spaces, action spaces, reward functions, and state transition probabilities. Unlike supervised learning’s reliance on labels, reinforcement learning uses reward signals. It encompasses value-based methods for discrete action spaces, using greedy strategies, and policy-based methods for continuous action spaces, producing continuous actions. To address the limitations of both, recent trends involve combining them into an actor–critic structure, enhancing learning outcomes.

#### 3.1.1. Deep Deterministic Policy Gradient Algorithm (DDPG)

DDPG, a seminal actor–critic algorithm, derives from DPG [14], featuring both policy and value functions. It is the first deep-reinforcement learning algorithm that uses the actor–critic framework, using neural networks to model DPG functions and address high-dimensional actions and states in continuous control. The training integrates deep Q-network techniques like target networks and experience replay, where the target network reduces overestimation and the replay buffer stores data. DDPG combines a policy network with a deep Q-network to output action values, necessitating the concurrent learning of both networks. The policy network (actor) generates deterministic actions, while the Q network (critic) evaluates these outputs to optimize action selection for maximum rewards. Bellman’s equation is employed to iteratively approximate the optimal action-value function.

(1) $$a^{*}(s)=ar\underset{a}{\mathrm{gmax}}Q^{*}(s,a)$$

Here, *s* represents the current state of the agent, *a* denotes the action produced by the policy network, and $Q^{*}\left(s,a\right)$ is the hypothetical optimal value function. This assumption is made because the real-world state space is vast, and the dynamics of the environment and reward function are highly nonlinear, rendering the computation of the optimal value function exceedingly difficult. The DDPG aims to identify the action that maximizes the value based on the fitted value function, thus optimizing the policy network’s gradient focus on maximizing this Q value. The strategy gradient formula is as follows:

(2) $$\begin{array}{ll} \nabla_{\theta^{\mu }}J & \approx E_{s_{t}∼\rho^{\beta }}\left[\nabla_{\theta^{\mu }}Q\left(s,a|\theta^{Q}\right){|}_{s=s_{t},a=\mu \left(s_{t}|\theta^{\mu }\right)}\right]\\ & =E_{s_{t}∼\rho^{\beta }}\left[\nabla_{a}Q\left(s,a|\theta^{Q}\right){|}_{s=s_{t},a=\mu \left(s_{t}\right)}\nabla_{\theta_{\mu }}\mu \left(s|\theta^{\mu }\right){|}_{s=s_{t}}\right] \end{array}$$

Here, $\theta^{\mu }$ represents the strategy network parameter, and $\theta^{Q}$ represents the value network parameter, with the Monte Carlo method used to obtain an unbiased estimate of the strategy gradient’s expectation. The value network optimization employs the reward *r* at time *t* and the discounted value $Q^{\prime }$ at time *t* + 1 to calculate the Q_target, aiming to align the Q network’s output closely with the Q_target. The root mean square error serves as the loss function [31], upon which the gradient descent is applied. The value network gradient formula is as follows:

(3) $$Q_{target}=r+\gamma Q^{\prime }\left(s^{\prime },\pi \left(s^{\prime }|\theta^{\mu^{\prime }}\right)|\theta^{Q^{\prime }}\right)$$

(4) $$\frac{\partial L\left(\theta^{Q}\right)}{\partial \theta^{Q}}=E_{s,a,r,s^{\prime }-D}\left[Q_{target}-Q\left(s,a|\theta^{Q}\right)\frac{\partial Q\left(s,a|\theta^{Q}\right)}{\partial \theta^{Q}}\right]$$

To make the Q_target more stable, DDPG builds the value network and the policy network with their target networks, Target_q and Target_p, respectively. The actor target network is responsible for selecting the optimal next action $A^{\prime }$ based on the next state $S^{\prime }$ sampled in the empirical playback pool, and the critic target network is responsible for calculating the $Q^{\prime }(S^{\prime },A^{\prime },\theta^{Q\prime })$. The parameters $\theta^{\mu^{\prime }}$ and $\theta^{Q^{\prime }}$ of the goal network are updated after a fixed period through soft updating.

To enhance the stability of the Q_target, DDPG constructs the value and policy networks along with their respective target networks, Target_q and Target_p. The actor target network selects the optimal next action $A^{\prime }$, based on the next state $S^{\prime }$ sampled from the empirical replay pool, while the critic target network calculates $Q^{\prime }(S^{\prime },A^{\prime },\theta^{Q\prime })$. The parameters $\theta^{\mu^{\prime }}$ and $\theta^{Q^{\prime }}$ of the target networks are updated periodically through soft updates, as such

(5) $$\begin{array}{c} \theta^{\mu^{\prime }}\leftarrow \tau \theta^{\mu }+\left(1-\tau \right)\theta^{\mu^{\prime }}\\ \theta^{Q^{\prime }}\leftarrow \tau \theta^{Q}+\left(1-\tau \right)\theta^{Q^{\prime }} \end{array}$$

The update coefficient *τ* is typically set to 0.1 or 0.01. Meanwhile, the DDPG adopts a deterministic strategy, unlikely to explore actions beyond the ones chosen to find useful learning signals. Therefore, adding noise to the selected actions enhances randomness in the learning process, enabling better exploration. However, random noise is not added to the actions predicted by the strategy network during the update process.

#### 3.1.2. Two-Delay Deep Deterministic Policy Gradient Algorithm (TD3)

Introduced by Scott Fujimoto et al. in 2018, the Twin-Delayed Deep Deterministic Policy Gradient (TD3) algorithm [17], with its actor–critic structure, excels in continuous control tasks. It improves upon the DDPG algorithm to tackle challenges in continuous action spaces.

**Figure 1:**
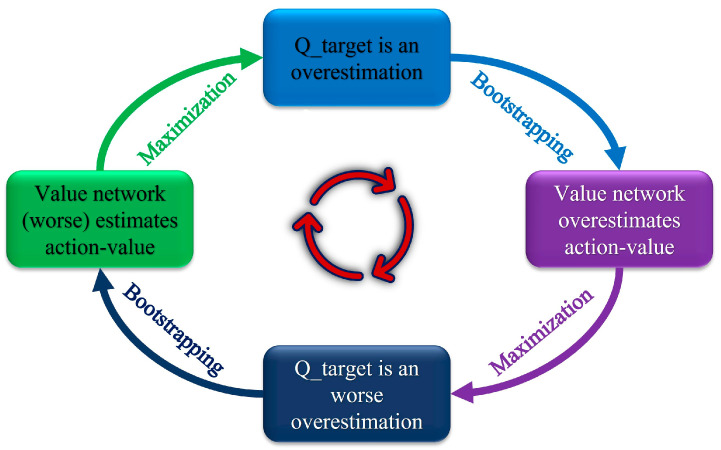
Schematic diagram of the overestimation process.

Overestimation of value functions is common in both discrete and continuous action learning, caused by maximization and bootstrapping, leading to error accumulation and suboptimal or divergent outcomes; Figure 1 illustrates the overestimation cycle process. Although DDPG addresses bootstrapping with a target network, it does not resolve overestimation from maximization. TD3 employs two sets of critic networks with identical architecture to address this, using the smaller output for target value calculation to guide learning. During learning, high variance in estimates from function approximation errors presents challenges, which TD3 mitigates by introducing slight random noise to target actions and calculating mean values over small batches.

(6) $$Q_{target}=r+\gamma \underset{i=1,2}{min}{Q_{i}}^{\prime }\left(s^{\prime },\pi \left(s^{\prime }|\theta^{\mu^{\prime }}\right)+\epsilon \right)$$

(7) $$\epsilon ∼\mathrm{clip}\left(N\left(0,\sigma \right),-c,c\right)$$

**Figure 2:**
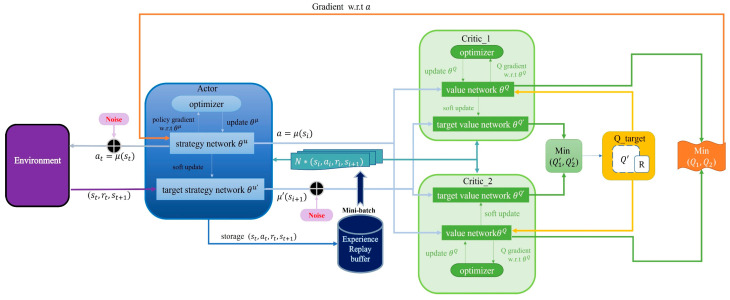
TD3 algorithm update flowchart.

The TD3 algorithm comprises six networks dedicated to intelligence learning. As illustrated in Figure 2, to circumvent the local optimum of the actor network, a delayed update strategy is employed where the actor is updated only after several updates of the critic. The actor target network’s update, in conjunction with the two critic target networks, mirrors the DDPG algorithm’s approach, employing a soft update method. An update coefficient τ weights the parameters of the old and new target networks before assigning them to the target network.

### 3.2. Improvement Methods

The TD3 algorithm, with its actor-critic architecture, is widely applied in robot navigation. While adept at managing high-dimensional states and actions and path planning in dynamic environments, it encounters challenges like low training efficiency and slow convergence. To overcome these, LP-TD3 integrates an LSTM module with a Prioritized Experience Replay mechanism [32,33], enhancing decision-making and navigation efficiency. Additionally, its reward function combines external and intrinsic curiosity rewards, boosting exploration and speeding up model training convergence.

#### 3.2.1. Navigation Principle of Inspection Robot Based on LP-TD3

Inspection robots synthesize historical data to develop optimal strategies for current situations. In partially observable Markov decision processes (POMDP) scenarios, such as obstacle avoidance, this study uses LSTM with prioritized experience replay to overcome the limitations of the TD3 network in long-term memory and learning efficiency. The robots constantly collect time series data, including LIDAR scans and camera images, through sensors. LSTM effectively captures long-term dependencies in these data, enhancing memory capabilities, and allowing for the retention of past observations and learning of environmental features for improved navigation decisions. The model also optimizes actions based on recent data, leading to smoother navigation paths.

**Figure 3:**
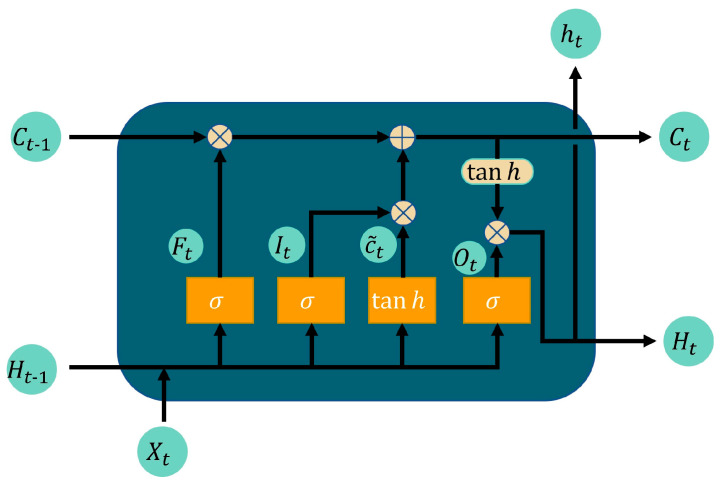
LSTM repetition module.

Figure 3 shows that LSTM contains not just a single network layer, but four interconnected neural network layers. Information transfer within LSTM’s repeating modules depends on the cell state, which can be regulated by a gate structure to add or remove information. An LSTM comprises three gates: the forgetting gate $F_{t}$, the input gate $I_{t}$, and the output gate $O_{t}$, which protects and controls the cell [27].

LSTMs can retain long-term information and recognize and utilize the information at any position within a sequence. This means that the network can access not only recent information, but also information processed long ago, thus overcoming short-term memory limitations. The “gate” structure design enables the network to filter sequence information, allowing it to determine post-training whether to retain certain information. In this paper, we fully leverage LSTM’s unique structure, enabling the model to retain experiential information throughout the navigation training, thereby enhancing its understanding of environmental dynamics.

#### 3.2.2. LP-TD3 Intelligent Body Inputs and Outputs

Network training relies on experience data generated from interactions with the environment, which includes the current state $s_{t}$, the action $a_{t}$ taken, the reward $r_{t}$, the next state $s_{t+1}$, and the termination flag, all stored in the experience replay pool. Originally, network training involved uniform sampling of a batch of experiences directly from the experience replay pool. However, the high correlation among experiences in the replay pool makes it challenging to distinguish their contributions to training in a uniform sampling approach. Therefore, only a simple replay of the experience cannot fulfill the need for efficient training.

The LP-TD3 algorithm introduced in this paper employs a prioritized experience replay mechanism, ranking the experiences in the replay pool by priority and sampling them accordingly. Specifically, the agent selects actions based on the environmental state, generating experience data that are stored in the replay pool. Subsequently, experiences in the replay pool are ranked and updated based on the discrepancy between target and actual values, using the TD error and the loss function. Following prioritization, the agent interacts with the environment to acquire new experiences, iterating this process in a continuous loop.

This study uses a two-wheeled differential speed robot for simulation experiments. The network model outputs continuous linear v and angular w velocities to control the robot’s forward movement and steering. The g and the previous action a serve as state input, enabling the neural network to gauge the robot’s speed and its distance from the target [34]. It is pertinent to highlight that g represents the Euclidean distance between the target coordinates ${T^{g}=(T}_{x}^{g},T_{y}^{g})$ and the current robot coordinates $T={(T}_{x}{,T}_{y})$, which is defined as follows:

(8) $$a=\left[v,w\right]$$

(9) $$s=\left[l,g,a\right]$$

(10) $$g=\sqrt{{{(T}_{x}-T_{x}^{g})}^{2}+{{(T}_{y}-T_{y}^{g})}^{2}}$$

In addition to incorporating the target point and the previous action as state inputs, the final state representation also includes laser data, resulting in a composite of one-dimensional vectors. The variable l represents 20-dimensional laser data, with the LIDAR’s scanning field of view set at 180 degrees, and evenly divided into 20 sections. During initialization, the start and end angles of each section are recorded. The smallest laser value in each section is recorded during interactions between the robot and its environment.

**Figure 4:**
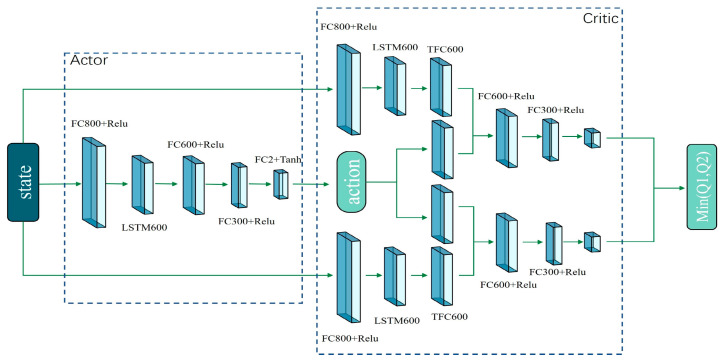
Network structure schematic.

To improve the model’s environmental understanding, this study enhances the original TD3 algorithm by incorporating a long–short-term memory (LSTM) module into both the actor and the critic networks. As illustrated in Figure 4, the actor network maps input states to action information, whereas the critic network evaluates the current strategy based on these inputs. The actor network comprises one LSTM layer and four fully connected layers, while the critic network contains one LSTM layer and five fully connected layers. The ReLU activation function is used during the forward-pass process.

Action information comprises a tuple of linear and angular velocities. Given the robot velocity’s specific constraints, the Tanh function is applied in the actor network’s final layer to restrict velocity information to the (−1, 1) interval. The model’s input is a state sequence comprising a batch of state information across multiple time steps. This state sequence is derived from sampling via a prioritized experience replay mechanism, with the sampled navigation experiences processed to yield a fixed-length, multi-time-step state sequence. Thus, the network model efficiently leverages empirical information throughout the navigation process, enabling quick decision-making in similar scenarios.

#### 3.2.3. Reward Function Design Based on ICM

The reward function, central to deep reinforcement learning, encompasses both intrinsic and extrinsic rewards to quantitatively evaluate an agent’s behavior in the environment. A good reward function provides robust feedback, ensuring similar actions receive comparable rewards, and motivating the agent to explore and avoid local optima. In navigation scenarios, the reward function’s role is to train the robot to complete its tasks. Typically, rewards or penalties are issued when the robot reaches the goal or experiences a collision. However, since reward signals are derived from experience, rewards tend to be sparse, leading to challenges in inefficiency and limited exploration ability during training. To address these issues, this paper introduces the Intrinsic Curiosity Module (ICM) as an intrinsic reward [35]. Combined with extrinsic rewards, this forms the comprehensive reward function module described herein.

(11) $$R=R^{\mathrm{i}}+R^{\mathrm{e}}$$

**Figure 5:**
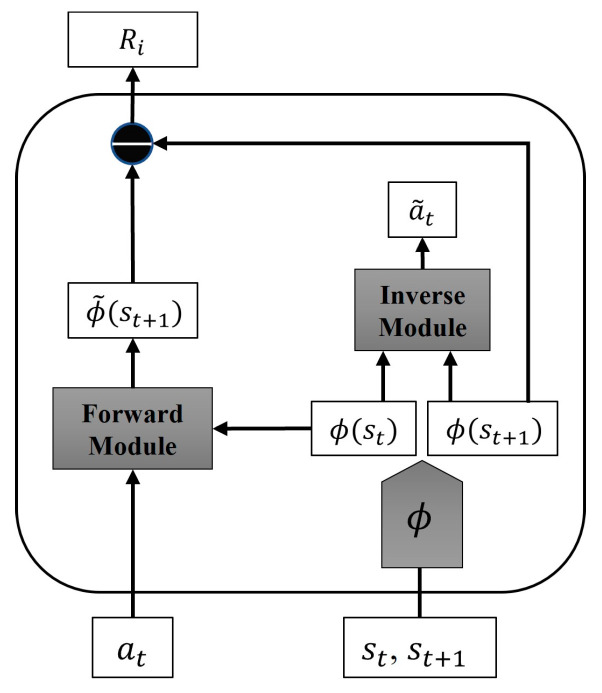
Structure of the intrinsic curiosity module.

Here, $R^{\mathrm{i}}$ denotes the intrinsic reward, and $R^{\mathrm{e}}$ represents the extrinsic reward, with the intrinsic reward $R^{\mathrm{i}}$ calculated using the Intrinsic Curiosity Module (ICM). The ICM is utilized to assess the agent’s ability to predict environmental changes from the current state, calculating the reward signal based on the prediction error’s magnitude. Figure 5 illustrates the ICM’s structure, comprising a feature extraction layer, a forward model, and a reverse model. The initial states $s_{t}$ and $s_{t+1}$ are encoded as $\phi \left(s_{t}\right)$ and $\phi \left(s_{t+1}\right)$ via the feature extraction layer. The inverse model predicts the agent’s action based on the current and subsequent states as follows:

(12) $${\tilde{a}}_{t}=g\left(\phi \left(s_{t}\right),\phi \left(s_{t+1}\right);\theta_{I}\right)$$

Here, $\theta_{I}$ represents the inverse model’s neural network parameter, and ${\tilde{a}}_{t}$ denotes the predicted action. The inverse model is refined by minimizing the discrepancy between the predicted and actual actions. Originally, the inverse model loss function in ICM was based on cross-entropy. However, in this study, due to the continuous action space, the loss function employs the mean squared deviation.

(13) $$L_{I}=\frac{1}{n}\sum_{i=1}^{n}{\left({\tilde{a}}_{t}-a_{t}\right)}^{2}$$

The forward model employs current actions and states to predict the subsequent state as follows:

(14) $$\tilde{\phi }\left(s_{t+1}\right)=f\left(\phi \left(s_{t}\right),a_{t};\theta_{F}\right)$$

Here, $\tilde{\phi }\left(s_{t+1}\right)$ represents the predicted state, and $\theta_{F}$ denotes the forward model’s neural network parameters. Training involves minimizing the loss function $L_{F}$ and using the prediction error between $\tilde{\phi }\left(s_{t+1}\right)$ and $\phi (s_{t+1})$ as the intrinsic reward, as follows:

(15) $$L_{F}=\frac{1}{2}{∥\tilde{\phi }\left(s_{t+1}\right)-\phi \left(s_{t+1}\right)∥}_{2}^{2}$$

(16) $$R^{\mathrm{i}}=\frac{1}{2}{∥\tilde{\phi }\left(s_{t+1}\right)-\phi \left(s_{t+1}\right)∥}_{2}^{2}$$

A large prediction error suggests that the agent’s predictions about environmental changes in a specific state are not accurate, resulting in a relatively large reward signal in such cases. The Intrinsic Curiosity Module generates an exploratory signal that significantly encourages the agent to explore unknown environments.

With the introduction of intrinsic rewards, the reward module now includes the sum of both intrinsic and extrinsic rewards. The composition of extrinsic rewards is as follows:

(17) $$R^{\mathrm{e}}=r^{t}+r^{v,w}$$

The reward $r^{t}$ comprises three elements: a reward for reaching the goal, a collision penalty, and a reward for approaching the goal. The reward $r^{v,w}$, based on linear and angular velocities, incentivizes the robot to advance and minimize rotational movements, aiming to avoid obstacles as effectively as possible.

(18) $$r^{t}=\left\{\begin{array}{c} r_{arrive}\ldots \ldots \ldots \ldots \ldots ifd_{t}<d_{a}\;\;\;\;\;\;\;\\ r_{collision}\ldots \ldots \ldots \ldots ifo_{min}<d_{c\;\;\;\;}\;\\ \xi \left(d_{t}-d_{t-1}\right)\ldots ..\ldots otherwise\;\;\; \end{array}\right.$$

(19) $$r^{v,w}=v-\left|w\right|-\eta d_{c}\cdots \cdots \eta \in \left(0,1\right)$$

The $d_{t}$ represents the current distance from the target point, $d_{a}$ is the threshold to determine if the target has been reached, $o_{min}$ the minimum value in the radar data, and $d_{c}$ the threshold for collision detection. ξ, the amplification coefficient for the reward related to the target point, is assigned a negative value. η, the obstacle avoidance coefficient increases in value as the proximity to an obstacle decreases.

## 4. Experiments

This section will validate the proposed LP-TD3 navigation method. This method necessitates extensive training data. Training in a real-world environment is time-consuming and poses a risk of damaging the equipment due to interactions between the agent and the environment. To rapidly assess the method’s effectiveness, training will be conducted in a simulation environment.

### 4.1. Experimental Parameters and Environment

The experiment will utilize two platforms, ROS and GAZEBO, with the open-source Turtlebot3 two-wheeled differential robot serving as the testbed. A computer equipped with an i7-11800H CPU, NVIDIA Geforce 3060 GPU, and 32 G RAM will serve as the training platform for this experiment. Hyperparameters were empirically set based on the performance capabilities of the training platform, as detailed in Table 1.

**Figure 6:**
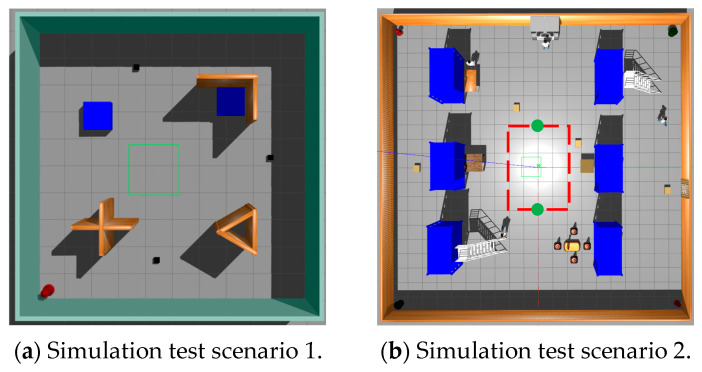
LP-TD3 simulation verification scenario.

This study undertakes a comparative analysis of the LP-TD3 navigation strategy and the original TD3 method, utilizing two simulated scenarios within the Gazebo environment, as illustrated in Figure 6. The first scenario assumes a static environment brimming with obstacles, such as fire hydrants, cardboard boxes, and cross-shaped walls. The second scenario, however, is more intricate and encompasses a larger map with the addition of dynamic pedestrian obstacles. Two such dynamic pedestrians are programmed to move in a clockwise direction along the trajectory delineated by the red dotted line in the figure, at a speed of 0.5 m/s. The green origin in the figure denotes the initial position of these two dynamic pedestrian obstacles in each episode. Moreover, four yellow cardboard boxes, each with dimensions of 0.5 m × 0.5 m × 0.5 m (as represented in the figure), are programmed to appear randomly at any location within this scene. This scene is designed to mimic a warehouse inspection environment, complete with warehouse shelves, randomly dispersed cardboard boxes, and designated maintenance areas. These scenarios partially replicate the real inspection environment and validate the comprehensiveness of the proposed navigation method.

**Figure 7:**
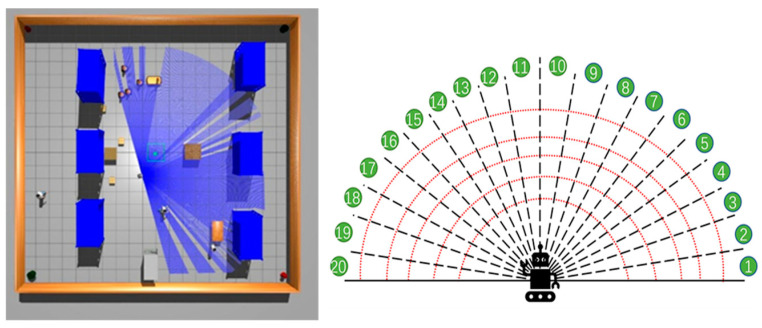
Schematic diagram of LP-TD3 status inputs.

The robot’s workflow involves gathering environmental data via external sensors and executing actions through its decision-making model based on these data. In this experiment’s simulation, the Turtlebot3 robot features a 16-line LIDAR with a 180-degree scan and a camera for visual inspection, with the LIDAR’s scan segmented into 20 dimensions. The blue ray in Figure 7 illustrates the visualization of the LIDAR scan. LIDAR scan data serve as state inputs to minimize discrepancies between the simulated and real environments. Navigation is achieved using 20-dimensional laser data, previous actions, and the target’s relative position. Each round randomly sets the robot’s initial position and the target point to avoid training overfitting. If the robot collides with an obstacle or misses the target within a round’s step limit, it ends the round to assess if it meets the decision-making model’s learning criteria before proceeding.

### 4.2. Analysis of Training Results

To compare the performance of the proposed method with the original method in different test environments, we define three performance metrics.

(1) Average reward $A_{r}$: $A_{r}$ is the average of the sum of episodes rewards when the model is evaluated.
(2) Collision rate $C_{r}$: $C_{r}$ is the ratio of total collisions to the number of episodes, assessing the model’s obstacle avoidance capability.
(3) Success rate $S_{u}$: $S_{u}$ measures the ratio of episodes where the robot successfully reaches the goal to the total number of episodes, evaluating the navigation performance.

**Figure 8:**
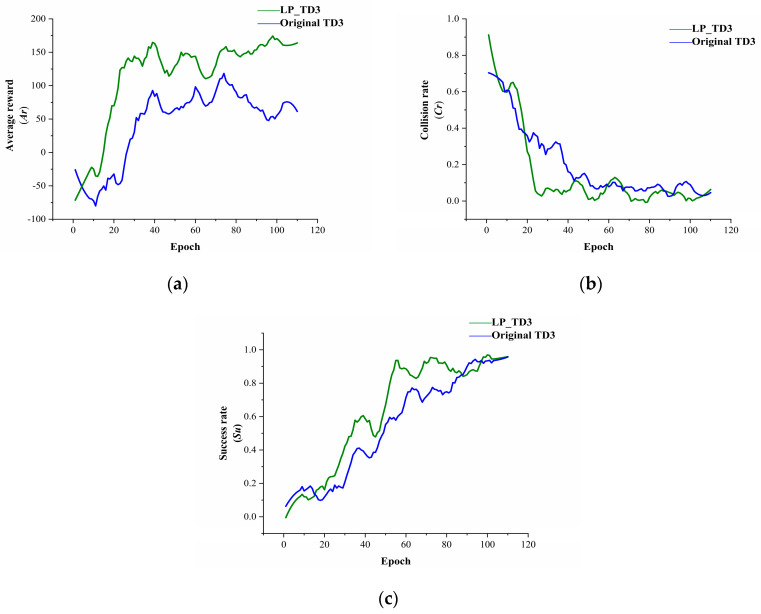
Indicator curves of different methods during training in scene 1. (**a**) Comparison of average reward between two methods. (**b**) Comparison of collision rate between two methods. (**c**) Comparison of success rate between two methods.

The results for scenario 1 are presented in Figure 8. Identical hyperparameters were used to train both algorithms. Training initiates upon the robot reaching the target or encountering a collision, with evaluations conducted every 5000 steps, repeated 15 times, to accurately showcase the model’s current performance. The figure shows smoothed evaluation results; the green curve represents the LP-TD3 algorithm, and the blue curve, the original TD3 algorithm.

In scenario 1, LP-TD3 significantly outperforms the original TD3 in average reward, collision rate, and success rate. The average reward curve indicates LP-TD3’s superior sample utilization efficiency, converging around the 20th Epoch compared to the 40th for TD3, showcasing faster convergence. Scene 1, with its small area and static obstacles like cross-shaped and L-shaped walls, can lead to local optima challenges for the robot. However, the experimental results suggest that the proposed strategy explores the environment more effectively than the original, successfully navigating to challenging targets, like those near cross-shaped and L-shaped walls.

**Figure 9:**
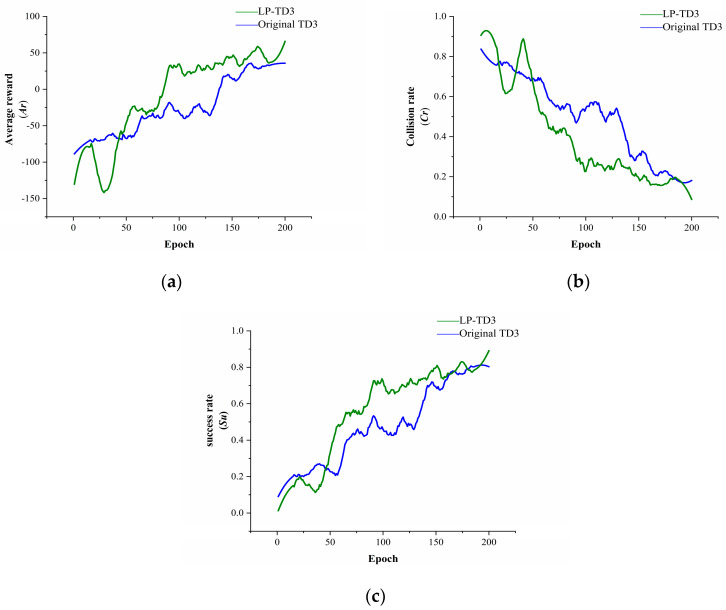
Indicator curves of different methods during training in scene 2. (**a**) Comparison of average reward between two methods. (**b**) Comparison of collision rate between two methods. (**c**) Comparison of success rate between two methods.

The training index curves for scenario 2, as illustrated in Figure 9, employ the same experimental parameters and methods as for scenario 1. Compared to scenario 1, scenario 2 presents a greater challenge with its larger size and complexity, especially due to the presence of dynamic obstacles that significantly hinder the robot’s ability to reach its destination. In such environments, robots are required to learn more sophisticated navigation strategies to effectively deal with these ever-present obstacles. Experimental results for scenario 2 indicate that, although both the proposed and original strategies necessitate additional time for environmental exploration, the proposed method surpasses the original in terms of model convergence speed, success rate, and collision rate. Initially, the LP-TD3 strategy, which emphasizes comprehensive exploration and leverages intrinsic curiosity rewards, exhibits lower performance metrics compared to the original TD3, as it facilitates the exploration of new states and prevents the robot from simply rotating in place, a limitation that the original TD3 strategy could not overcome. However, by the 50th epoch, LP-TD3 begins to outperform the original strategy, and by the 100th epoch, it adeptly navigates around obstacles, successfully reaching the destination, and completing the navigation task.

### 4.3. Analysis of Evaluation Results

**Figure 10:**
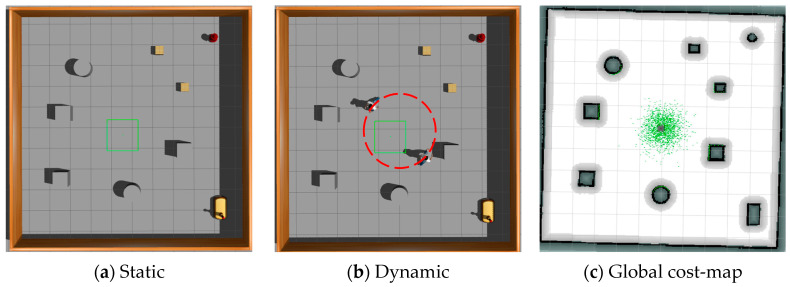
Simulation test environment.

To assess the effectiveness of the local planning algorithm proposed in this study, we constructed a test environment in GAZEBO. As depicted in Figure 10, the test environment comprises a 10 × 10 square meter area featuring multiple obstacles. The test environment is divided into two levels to evaluate the algorithm’s performance. The first level features a static environment, while the second introduces a dynamic setting with two pedestrians moving counterclockwise, as indicated by the red dotted line in the figure, at a speed of 0.6 m/s. For the test task, we designated 10 sequential points for the planner; the robot must navigate these sub-objectives in sequence while avoiding obstacles.

To demonstrate the distinctions between the proposed method and traditional approaches, we will compare it with the original TD3 algorithm and the move_base. The move_base is a fundamental navigation component offered within the Robot Operating System (ROS). This intricate component is responsible for executing paths that are generated collaboratively by both the global and local planners, thereby performing motion control. In addition to this, move_base also incorporates local position estimation coupled with dynamic map updates to enhance navigation capabilities. In the comparative experiment conducted, the default global planner of move_base, which is based on Dijkstra’s algorithm, is utilized alongside the local planner that employs the Dynamic Window Approach (DWA) algorithm. The specific parameter settings utilized for this experiment are detailed in Appendix A.

**Figure 11:**
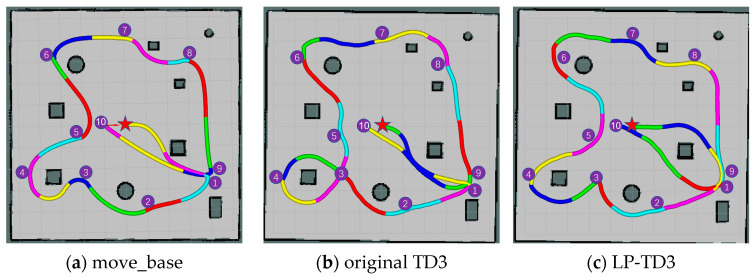

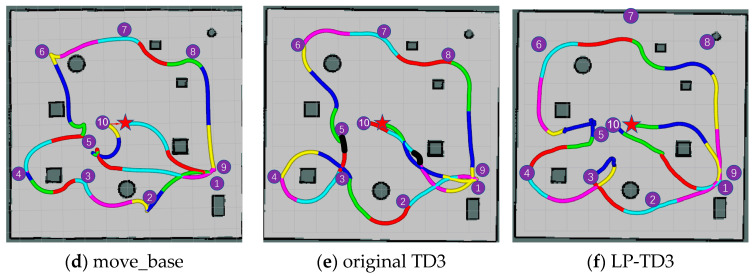
Path trajectory in the virtual test environment. (**a**–**c**) show static environment paths and (**d**–**f**) show dynamic environment paths.

The move_base requires construction of a global map for path planning, whereas the proposed method uses this map solely for visual display. The navigation path results for the three methods in both static and dynamic environments are depicted in Figure 11, marked by a red pentagram indicating the robot’s starting point. To effectively illustrate the impact of varying methods under identical experimental parameters, the robot’s position was recorded at 5 s intervals. As depicted in Figure 11, line segments of diverse colors signify the paths traversed by the robot during these 5 s intervals. This color-coded representation facilitates a clear understanding of the robot’s movement under different methods.

We selected two metrics to evaluate the planners: the time taken by the robot to complete the navigation task and the total distance traveled. Within the testing environment, each method was executed 10 times. Figure 11 demonstrates one of the generated paths for each method. The generated experimental data are presented in Table 2, which details the test results for both static and dynamic environments.

In the static test environment, Figure 11a–c displays the trajectory plots for the navigation tasks, showing that all methods can complete the tasks without collisions. According to Table 2, the conventional navigation method, move_base, reduces the cumulative distance traveled by 15.13% and 9.57% compared to the original TD3 and LP-TD3 algorithms, respectively, suggesting that the paths generated using deep reinforcement learning-based methods are not optimal. In a static environment, while the method proposed in this paper does not surpass the performance of the classical algorithm, it indeed shows a marked improvement over the original TD3.

In a dynamic environment, the move_base successfully completes the navigation task most of the time. However, in certain instances, such as during the robot’s journey from navigation point 9 to navigation point 10 as illustrated in Figure 11d, the robot encountered challenges when avoiding pedestrians. Specifically, while the robot managed to plan a route to circumvent the first pedestrian it encountered, it struggled to react in time to a second approaching pedestrian. This delay resulted in the robot remaining stationary until the pedestrian was very close, at which point the robot hastily reversed and altered its path. The original TD3 algorithm’s inability to effectively navigate around dynamic pedestrians is highlighted by the interruption indicated by a black line segment in Figure 11e, requiring human intervention to resume the navigation task. In contrast, our proposed LP-TD3 method, presented in Figure 11f, successfully completes the navigation task under similar circumstances. For instance, during the robot’s movement from navigation point 5 to navigation point 6, as pedestrians gradually approached, the robot swiftly opted to turn left to avoid them. The experimental results demonstrate that LP-TD3 is capable of successfully completing navigation tasks even in the absence of a map.

## 5. Conclusions

This study introduces a mapless navigation solution for indoor inspection scenarios, addressing the poor performance of current navigation methods via deep reinforcement learning. Building on the existing TD3 algorithm, we propose the LP-TD3 algorithm, which combines intrinsic curiosity rewards with extrinsic rewards to motivate the robot to explore its environment. LP-TD3 equips the robot with both long-term and short-term memory modules, enhancing learning from beneficial navigational experiences through a prioritized experience replay mechanism. The model inputs comprise the robot’s current actions, LiDAR scan data, and its relative position to the target, offering comprehensive learning data. In the training phase, the algorithm surpasses the original TD3 in average reward, collision rate, success rate, and achieves faster convergence. Simulation tests demonstrate that the LP-TD3 local planning algorithm enables efficient navigation amidst both static and dynamic obstacles. Therefore, the method proposed in this paper is applicable to inspection scenarios, such as in factories, to facilitate smarter and safer production activities. This paper focuses on enhancing learning efficiency and addressing the reward sparsity issue of the original method. Future research will aim at deploying these learning algorithms on actual detection robots, enabling ongoing post-deployment learning for optimal real-world performance and long-range navigation path optimization.

## Figures and Tables

**Table 1:** Experimental parameters.

Parameters	Value
Learn rate	0.001
Discount factor	0.999
Batch size	32
Soft update	0.005
Initial exploration	1
Final exploration	0.1
*r_arrive_*	120
*r_collision_*	−120
ξ	−5

**Table 2:** Indicator values for different methods.

Method	Distance (Static)	Time (Static)	Distance (Dynamic)	Time (Dynamic)
Move_base	41.71 m	95.66 s	46.13 m	135.63 s
Original TD3	48.02 m	105.70 s	48.96 m	133.30 s
LP-TD3	45.70 m	97.13 s	47.71 m	120.98 s

## Data Availability

The data presented in this study are available on request from the corresponding author.

## Appendix A

**Table A1:** The move_base navigation package global parameters.

Global Planner Parameters	Value
shutdown_costmaps	False
controller_frequency	10.0
planner_patience	5.0
controller_patience	15.0
conservative_reset_dist	3.0
planner_frequency	5.0
oscillation_timeout	10.0
oscillation_distance	0.3

**Table A2:** The move_base navigation package local parameters.

Local Planner Parameters	Value
max_vel_x	1.0
min_vel_x	−1.0
max_vel_y	0.0
min_vel_y	0.0
max_vel_trans	1.0
min_vel_trans	−1.0
max_vel_theta	1.0
min_vel_theta	−1.0
acc_lim_x	2.5
acc_lim_y	0.0
acc_lim_theta	3.2
xy_goal_tolerance	0.05
yaw_goal_tolerance	0.17
latch_xy_goal_tolerance	false
sim_time	2.0
vx_samples	20
vy_samples	0
vth_samples	40
controller_frequency	10.0
path_distance_bias	32.0
goal_distance_bias	20.0
occdist_scale	0.02
forward_point_distance	0.325
stop_time_buffer	0.2
scaling_speed	0.25
max_scaling_factor	0.2
oscillation_reset_dist	0.05
